# Tumor Necrosis Factor-α Induced Protein 8 Polymorphism and Risk of Non-Hodgkin’s Lymphoma in a Chinese Population: A Case-Control Study

**DOI:** 10.1371/journal.pone.0037846

**Published:** 2012-05-29

**Authors:** Yan Zhang, Meng-Yun Wang, Jing He, Jiu-Cun Wang, Ya-Jun Yang, Li Jin, Zhi-Yu Chen, Xue-Jun Ma, Meng-Hong Sun, Kai-Qin Xia, Xiao-Nan Hong, Qing-Yi Wei, Xiao-Yan Zhou

**Affiliations:** 1 Department of Medical Oncology, Fudan University Shanghai Cancer Center, Shanghai, China; 2 Department of Oncology, Shanghai Medical College, Fudan University, Shanghai, China; 3 Cancer Research Laboratory, Fudan University Shanghai Cancer Center, Shanghai, China; 4 Ministry of Education Key Laboratory of Contemporary Anthropology and State Key Laboratory of Genetic Engineering, School of Life Sciences, Fudan University, Shanghai, China; 5 Fudan-Taizhou Institute of Health Sciences, Taizhou, Jiangsu, China; 6 Department of Radiation Oncology, Fudan University Shanghai Cancer Center, Shanghai, China; 7 Department of Pathology, Fudan University Shanghai Cancer Center, Shanghai, China; 8 Department of Epidemiology, the University of Texas MD Anderson Cancer Center, Houston, Texas, United States of America; University of Navarra, Center for Applied Medical Research, Spain

## Abstract

**Background:**

Non-Hodgkin’s lymphoma (NHL) has been reported to be associated with autoimmune and pro-inflammatory response, and genetic polymorphisms of candidate genes involved in autoimmune and pro-inflammatory response may influence the susceptibility to NHL. To evaluate the role of such genetic variations in risk of NHL, we conducted a case-control study of 514 NHL patients and 557 cancer-free controls in a Chinese population.

**Method:**

We used the Taqman assay to genotype six potentially functional single nucleotide polymorphisms (SNPs) in six previously reported inflammation and immune-related genes (*TNF* rs1799964T>C, *LTA* rs1800683G>A, *IL-10* rs1800872T>G, *LEP* rs2167270G>A, *LEPR* rs1327118C>G, *TNFAIP8* rs1045241C>T). Logistic regression models were used to estimate odds ratios (ORs) and 95% confidence intervals (95% CI).

**Results:**

We observed a significantly increased risk of NHL associated with the *TNFAIP8* rs1045241C>T polymorphism (adjusted OR = 3.03; 95% CI = 1.68–5.45 for TT vs. CC and adjusted OR = 2.03; 95% CI = 1.53–2.69 for CT/TT vs. CC). The risk associated with the T allele was more evident in subgroups of 40–60 year-old, non-smokers or light-smokers (less than 25 pack-years), and subjects with normal weight or overweight. Risk for both B and T cell non-Hodgkin’s lymphoma was elevated for CT/TT genotypes (adjusted OR = 1.95, 95% CI = 1.41–2.70 for B cell NHL and adjusted OR = 2.22, 95% CI = 1.49–3.30 for T cell NHL), particularly for DLBCL (adjusted OR = 2.01, 95%CI = 1.41–2.85) and FL (adjusted OR = 2.53, 95% CI = 1.17–5.45). These risks were not observed for variant genotypes of other five SNPs compared with their common homozygous genotypes.

**Conclusions:**

The polymorphism of *TNFAIP8* rs1045241C>T may contribute to NHL susceptibility in a Chinese population. Further large-scale and well-designed studies are needed to confirm these results.

## Introduction

Non-Hodgkin’s lymphoma (NHL) incidence rates have been increasing in both developed and developing countries with about 355,900 new cases in the world annually [1]. In China, the most common subtype of NHL is diffuse large B cell lymphoma (DLBCL), whereas follicular lymphoma (FL) is less common than in Western countries. Extranodal lesions and T/NK cell NHLs (eg. Extranodal NK/T-cell lymphoma) appear to be more common in China [2]. However, the exact causes of NHL remain largely unknown. Some evidence has showed that immune dysfunction may be one of the risk factors [3], and single nucleotide polymorphisms (SNPs) in immune and inflammatory response genes may play an important role in lymphomagenesis [4], [5], [6], [7].

*TNF/LTA* and *IL-10* genes code for immunoregulatory cytokines that can mediate inflammation, apoptosis and Th1/Th2 balance [6], [7], and they may be good candidate genes for studying lymphomagenesis. The cytokines TNF-α and LT-α are thought to influence lymphomagenesis through up-regulation of pro-inflammatory and anti-apoptotic signals, possibly via the NK-κB pathway [5]. Some evidence also showed that polymorphisms in *IL-10* may regulate TNF-α levels and thus contribute to activation of the NK-κB pathway [8]. A pooled analysis including 7,999 NHL cases and 8,452 controls from 14 case-control studies was carried out by InterLymph Consortium, which showed that *LTA* 252A?G (rs909253), *IL-1*-3575T?A (rs1800890), and particularly *TNF*-308G?A (rs1800629) were associated with an increase risk of DLBCL in non-Hispanic white populations [9]. Purdue et al. reported that *IL-10*-3575T?A and *TNF*-863C?A (rs1800630) were associated with an elevated risk of DLBCL in an Australian case-control study [5]. A recent genome-wide association study (GWAS) of FL has identified additional two variants in the 6p21 chromosomal region [10], which is the *TNF* gene location, suggesting that genetic variants in these regions may influence NHL susceptibility.

The tumor necrosis factor-α induced protein 8 (TNFAIP8) family are newly identified proteins that are important for inflammation and immune homeostasis [11]. They play roles in anti-inflammation by negatively regulating T cell receptor (TCR) and Toll-like receptor (TLR) signaling [12]. But the association between *TNFAIP8* polymorphisms and NHL risk has not been reported so far, particularly in Chinese populations.

The circulating levels of adipocytokines, including adiponectin, resistin and leptin may also alter immune system function and chronic inflammatory response. Leptin has pro-inflammatory properties and stimulates the growth of certain cancer cells as well as circulating pro-inflammatory cytokines, such as TNF-α and interleukin [13]. Associations between NHL and polymorphisms in the leptin (*LEP*) and leptin receptor (*LEPR*) gene have also been reported. Skibola et al. found that the *LEP*19G allele was associated with an increased risk of NHL, particularly FL [14]. A similar result was reported by a UK study, in which the *LEPR* 223ArgArg genotype was shown to be associated with an increased risk of FL among women [15].

To test the hypothesis that polymorphisms in inflammation and immune-related genes (such as *TNF, LTA, IL-10, LEP, LEPR* and *TNFAIP8*) may be associated with susceptibility to NHL, we conducted a case-control study in a Chinese population and genotyped six potentially functional SNPs in the above-mentioned candidate genes.

## Materials and Methods

### Ethics Statement

The study was approved by the Institutional Review Board of Fudan University Shanghai Cancer Center. Participation was voluntary. All participants singed a written informed consent, and all clinical investigation was conducted according to the principles expressed in the Declaration of Helsinki consent.

### Study Population

The study population was identified from histologically confirmed NHL cases diagnosed and treated between June 2005 and September 2011 at Fudan University Shanghai Cancer Center. All patients came from the Eastern China, including Shanghai, Jiangsu Province and the surrounding regions. Enrollment criteria included the following: Chinese Han ethnicity, HIV antibody negative, and without a diagnosis of second primary malignancy. All cases were classified and reviewed according to the 2008 WHO classification of tumors of haematopoietic and lymphoid tissues [16]. Of the 732 eligible cases, 514 (70.2%) consented to participate in the study and provided blood samples. Additionally, 557 cancer-free control subjects were all selected from Taizhou Longitudinal Study (TZL) at the same time period. All the control subjects were frequency matched to the cases on age (according to cases’ age groups by every 5 years) and sex. The cancer-free controls were genetically unrelated ethnic Han Chinese who were not selected from family members of patients and had no blood relationship. All participants were all from Eastern China, and there was a major geographical overlap between the cases and controls. TZL was initiated in July, 2007, in Taizhou, Jiangsu province of China, as described previously [17].

### Data Collection

After signing a written informed consent, all cases and controls were asked to provide a blood sample of about 2 ml and completed a questionnaire. Cases were asked about information including age, sex, ethnicity, status of smoking and drinking, the height and weight before diagnosis. The controls were also asked to recall the same questions prior to the recruitment date. A total of 514 cases and 557 controls provided blood samples, but 121 (23.5%) cases and 22 (3.9%) controls did not provide information about smoking and drinking, and 91 (17.7%) cases failed to give the exact information of height and weight before diagnosis.

### SNP Selection

We first screened five frequently reported (*TNF*, *LTA*, *IL-10*, *LEP*, *LEPR*) and one newly identified (*TNFAIP8*) inflammation and immune-related genes from published papers. We then searched SNPs in these six genes by NCBI dbSNP database (http://www.ncbi.nlm.nih.gov/) for all populations, and used HapMap database (http://hapmap.ncbi.nlm.nih.gov/) to focus on CHB (Han Chinese in Beijing, China) population. SNPinfo (http://snpinfo.niehs.nih.gov/) website was used as a tool to predict SNP functions. Potentially functional SNPs were defined to fit at least three of following four criteria: (1) SNPs located at the two ends of these genes, such as 5′ near gene, 5′ untranslated region (UTR), 3′ near gene or 3′UTR; (2) the minor allele frequency (MAF) was≥5% in the Hapmap CHB population. (3) SNP variation may affect transcription factor binding sites (TFBS) activity in the putative promoter region (here defined as 2-kb upstream from the first exon). (4) SNP may affect the microRNA-binding sites activity. Then eight SNPs were identified (*TNF* rs1799964T>C, *LTA* rs1800683G>A, *IL-10* rs1800872T>G, *LEP* rs2167270G>A, *LEP* rs4728096 T>C, *LEPR* rs1327118C>G, *TNFAIP8* rs1045241C>T, *TNFAIP8* rs11064A>G). However, the linkage disequilibrium (LD) analysis revealed that *LEP* rs2167270G>A and *LEP* rs4728096 T>C were in quite high LD (r^2^ = 0.93), and *TNFAIP8* rs1045241C>T and rs11064A>G were also in LD with r^2^ = 0.85. As a result, we selected following six potentially functional and representative SNPs (*TNF* rs1799964T>C, *LTA* rs1800683G>A, *IL-10* rs1800872T>G, *LEP* rs2167270G>A, *LEPR* rs1327118C>G, *TNFAIP8* rs1045241C>T) for further genotyping.

### Genotyping

Genomic DNA was extracted from each blood sample by using the Qiagen Blood DNA Mini Kit (Qiagen Inc., Valencia, CA) according to the manufacturer’s instructions. DNA purity and concentration were determined by spectrophotometric measurement of absorbance at 260 and 280 nm by a UV spectrophotometer (Nano Drop Technologies, Inc., Wilmington, DE) and all are qualified.

All TaqMan assays for this study including the pre-designed SNP-genotyping assay mix containing PCR primers and probes were purchased from ABI (Applied Biosystems, Foster City, CA). Genotyping were conducted on the ABI 7900HT detection system (Applied Biosystems). To ensure the accuracy of genotyping results, four negative controls (no DNA) and four duplicated samples were included in each of the 384-well plates for the quality control. The assays were repeated for 5% of the samples, and the results were 100% concordant. The analyzed fluorescence results were then auto-called into the genotypes using the built-in SDS2.2 software of the system.

### Statistical Analysis

BMI was calculated as weight (kg) divided by the square of the height (m). In this study, we used the BMI cutoff points as suggested by the Cooperative Meta-Analysis Group of Working Group on Obesity in China [18]. If BMI<18.5 kg/m^2^, the individuals were defined as lower than normal weight, 18.5≤BMI≤24.0 kg/m^2^ was defined as normal weight and BMI>24.0 kg/m^2^ was defined as overweight. Smoking status was divided into smokers and non-smokers by whether or not they had smoked for more than one year. Those who drank alcoholic beverages at least once a week for one year or more were defined as alcohol users, while the others were non-users. Differences in the distributions of the alleles and genotypes as well as demographic characters, smoking status, alcohol use and BMI between the cases and controls were evaluated by the Chi-square test. The Hardy–Weinberg equilibrium (HWE) of genotype distribution in the controls was tested by a goodness-of-fit Chi-square test. Unconditional univariate and multivariate logistic regression models were applied to calculate crude and adjusted odds ratios (ORs) and 95% confidence intervals (95% CI). We used the multiple imputation (MI) method by SAS 9.1 software to handle missing covariate information. All covariates were imputed, when calculated adjusted ORs, according to the distributions of observed values of either cases or controls. All statistical tests were two-sided, and P<0.05 was considered statistically significant. All analyses were performed using SAS Software, version 9.1 (SAS Institute, Cary, NC).

## Results

### Characteristics of the Study Population

There were 514 NHL cases and 557 cancer-free controls included in this study, whose DNA samples were available. The frequency distributions of demographic and some selected characteristics of the participants are shown in **Table 1** **.** There was no statistical difference in the distributions of age and sex between cases and controls because of frequency matching by design. The mean age was 49.3 years for the cases (±14.1; range, 15–85) and 49.6 years for the controls (±13.5; range, 20–85; *P* = 0.895), and 64.0% of the cases and 63.4% of the controls were male (*P* = 0.830). The controls were more likely to be smokers and alcohol users (*P*<0.0001 and *P* = 0.0001, respectively) and have higher BMI (*P*<0.0001) compared with the cases. Consequently, smoking status, alcohol use and BMI were adjusted for in the subsequent multivariate logistic regression analyses. Of the 514 cases, 336 (65.4%) were B cell lymphoma, and 178 (34.6%) were T cell and natural killer (NK) cell lymphoma. After stratified, 233 (45.3%), 52 (10.1%), 51 (9.9%), 146 (28.4%), 32 (6.2%) were DLBCL, FL, other B cell lymphoma, NK/T cell lymphoma, and other T cell lymphoma, respectively. Of all cases, 322 (62.6%) had an Ann Arbor Stage of I–II and 192 (37.4%) had a later Ann Arbor Stage of III–IV.

**Table 1:** Characteristics of Non-Hodgkin’s lymphoma cases and cancer-free controls.

Variables	Cases No. (%)	Controls No. (%)	*P-*value^a^
All subjects	514 (100)	557 (100)	
Age (years)			0.895
Median (Range)	50.5 (15–85)	51.0 (20–85)	
<40	129 (25.10)	131 (23.52)	
40–60	269 (52.33)	299 (53.68)	
>60	116 (22.57)	127 (22.80)	
Sex			0.830
Male	329 (64.0)	353 (63.4)	
Female	185 (36.0)	204 (36.6)	
Smoking status			<0.0001
Smoker	101 (25.7)	232 (43.4)	
Non-smoker	292 (74.3)	303 (56.6)	
Missing	121	22	
Pack-years			<0.0001
0	292 (74.3)	302 (56.4)	
0–25	64 (16.3)	171 (32.0)	
>25	37 (9.4)	62 (11.6)	
Missing	121	22	
Alcohol use			0.0001
Yes	58 (14.8)	134 (25.0)	
No	335 (85.2)	401 (75.0)	
Missing	121	22	
BMI (kg/m^2^)			<0.0001
Median	22.8	23.7	
<18.5	29 (6.9)	21 (3.8)	
18.5–24.0	253 (59.8)	273 (49.0)	
>24.0	141 (33.3)	263 (47.2)	
Missing	91	0	
Subtype			
B cell lymphoma	336 (65.4)	–	
DLBCL	233 (45.3)	–	
FL	52 (10.1)	–	
Other B cell NHL	51 (9.9)	–	
T cell lymphoma	178 (34.6)	–	
NK/T	146 (28.4)	–	
Other T cell NHL	32 (6.2)	–	
Ann Arbor Stage		–	
I–II	322 (62.6)	–	
III–IV	192 (37.4)	–	

a ***P*** value of the comparison with a two-sided Chi-square test.

### Association between Selected SNPs and Risk of NHL

Genotype distributions of the selected six SNPs in cases and controls and their associations with NHL risk are presented in **Table 2**. All observed genotype distributions among controls were in agreement with the Hardy–Weinberg equilibrium (*P* = 0.285 for rs1799964, *P* = 0.777 for rs180683, *P* = 0.872 for rs1800872, *P* = 0.733 for rs2167270, *P* = 0.559 for rs1327118, *P* = 0.420 for rs1045241). Significant difference in the genotype frequencies was observed between the cases and controls for *TNFAIP8* rs1045241C>T (*P*<0.0001). When the rs1045241CC genotype was used as the reference, the CT heterozygous, TT homozygous and combined CT/TT genotypes were associated with significantly increased risk of NHL (adjusted OR = 1.88; 95% CI = 1.39–2.53 for CT heterozygous genotype, adjusted OR = 3.03; 95% CI = 1.68–5.45 for TT homozygous genotype, adjusted OR = 2.03; 95% CI = 1.53–2.69 for CT/TT genotype) after adjustment for age, sex, BMI, smoking and drinking status. However, no significantly altered NHL risk was found for variant genotypes of the other five SNPs compared with their common genotypes. We also evaluated the combined effect of all the six SNPs. We had divided the subjects into seven groups according to the number of combined variant genotypes. The “0” risk genotype group was used as the reference, and unconditional logistic regression models were applied to calculate OR and 95%CI for each group. But we did not find any significant association between the combined effect of these six SNPs and risk of NHL (data not shown).

**Table 2:** Genotypes distributions of the selected functional polymorphisms among NHL cases and cancer-free controls and their associations with NHL risk.

Genotyping	Cases			Controls			*P*	Crude OR (95% CI)	*P*	Adjusted OR^a^ (95% CI)	*P*
	n		%	n		%					
*TNF* rs1799964T>C							0.590^b^				
TT	315	61.3		358	64.3			1.00		1.00	
CT	183	35.6		182	32.7			1.14 (0.89–1.48)	0.305	1.10 (0.82–1.47)	0.531
CC	16	3.1		17	3.0			1.07 (0.53–2.15)	0.850	0.75 (0.33–1.70)	0.495
CT/CC	199	38.7		199	35.7		0.312^c^	1.14 (0.89–1.46)	0.312	1.06 (0.80–1.41)	0.667
*LTA* rs1800683G>A							0.238^b^				
GG	125	24.3		161	28.9			1.00		1.00	
AG	275	53.5		280	50.3			1.26 (0.95–1.68)	0.108	1.30 (0.94–1.81)	0.112
AA	114	22.2		116	20.8			1.27 (0.89–1.79)	0.185	1.30 (0.88–1.94)	0.188
AG/AA	389	75.7		396	71.1		0.090^c^	1.26 (0.96–1.66)	0.090	1.30 (0.96–1.78)	0.094
*IL-10* rs1800872T>G							0.279^b^				
TT	226	44.0		269	48.3			1.00		1.00	
GT	228	44.3		235	42.2			1.15 (0.90–1.49)	0.267	1.23 (0.92–1.65)	0.158
GG	60	11.7		53	9.5			1.35 (0.89–2.03)	0.154	1.46 (0.92–2.31)	0.106
GT/GG	288	56.0		288	51.7		0.156^c^	1.19 (0.94–1.51)	0.156	1.28 (0.97–1.68)	0.083
*LEP* rs2167270G>A							0.801^b^				
GG	322	62.6		338	60.7			1.00		1.00	
AG	166	32.3		190	34.1			0.92 (0.71–1.19)	0.512	0.93 (0.69–1.25)	0.631
AA	26	5.1		29	5.2			0.94 (0.54–1.63)	0.829	0.92 (0.48–1.76)	0.792
AG/AA	192	37.4		219	39.3		0.509^c^	0.92 (0.72–1.18)	0.509	0.93 (0.70–1.23)	0.607
*LEPR* rs1327118C>G							0.769^b^				
CC	390	75.9		412	74.0			1.00		1.00	
CG	116	22.6		136	24.4			0.90 (0.68–1.20)	0.472	0.93 (0.67–1.29)	0.645
GG	8	1.5		9	1.6			0.94 (0.36–2.46)	0.898	1.19 (0.39–3.68)	0.760
CG/GG	124	24.1		145	26.0		0.472^c^	0.90 (0.69–1.19)	0.473	0.94 (0.68–1.29)	0.705
*TNFAIP8* rs1045241C>T							**<0.0001** b				
CC	293	57.0		381	68.4			1.00		1.00	
CT	180	35.0		156	28.0			**1.50 (1.15–1.95)**	**0.003**	**1.88 (1.39–2.53)**	**<0.0001**
TT	41	8.0		20	3.6			**2.67 (1.53–4.65)**	**0.0005**	**3.03 (1.68–5.45)**	**0.0002**
CT/TT	221	43.0		176	31.6		**0.0001** c	**1.63 (1.27–2.10)**	**0.0001**	**2.03 (1.53–2.69)**	**<0.0001**

Statistically significant results (*P*<0.05) are highlighted in bold.

a ORs were obtained from logistic regression models with adjustment for age, sex, smoking status, alcohol use and BMI.

b Two-sided Chi-square test for distribution of three genotypes.

c Two-sided Chi-square test for distribution of combined genotypes.

### Stratified Analysis

We further evaluated the association between the six candidate SNPs and risk of NHL by subgroups of age, sex, smoking status, alcohol use, BMI, common subtypes and Ann Arbor stage. No statistical significances were found for the SNPs except *TNFAIP8* rs1045241 C>T. The stratified analysis results of *TNFAIP8* rs1045241 C>T are presented in **Table 3**. In general, an increased risk associated with rs1045241 CT/TT genotypes was more evident in subgroups of 40–60 year-old individuals (adjusted OR = 2.99, 95% CI = 2.03–4.43), non-smokers (adjusted OR = 1.90, 95% CI = 1.35–2.68) or smoked less than 25 pack-years (adjusted OR = 2.45, 95% CI = 1.32–4.54), and normal weight (adjusted OR = 1.76, 95% CI = 1.23–2.52) or overweight groups (adjusted OR = 2.07, 95% CI = 1.32–3.25). Moreover, the patients with rs1045241 CT/TT genotypes were associated with risk of both B and T cell lymphoma (adjusted OR = 1.95, 95% CI = 1.41–2.70 and adjusted OR = 2.22, 95% CI = 1.49**–**3.30, respectively). After stratifying by histological subtypes, we observed increased risk for DLBCL (adjusted OR = 2.01, 95%CI = 1.41–2.85) and FL (adjusted OR = 2.53, 95%CI = 1.17–5.45), but no significant association was found for NK/T cell lymphoma (adjusted OR = 0.79, 95%CI = 0.27–2.33). However, after we verified homogeneity assumption by using a Chi square-based Q-test. The results indicated that an increased NHL risk associated with CT/TT genotypes was particularly more pronounced only in subgroups of 40–60 year-old (*P* = 0.03), and patients with a later Ann Arbor stage of III–IV (*P* = 0.04).

**Table 3:** Stratification analysis of the association between *TNFAIP8* rs1045241C>T and NHL risk.

Variables	CT+TT (cases/controls)		CC (cases/controls)		P^a^	OR (95%CI)		P^c^
	n	%	n	%		Crude	Adjusted^b^	
All subjects	221/176	43.0/31.6	293/381	57.0/68.4	**0.0001**	**1.63(1.27**–**2.10)**	**2.03(1.53**–**2.69)**	
Age (year)								**0.030**
<40	56/42	43.3/32.1	73/89	56.6/67.9	0.259	1.63(0.98–2.70)	1.43(0.77–2.67)	
40–60	119/81	44.2/27.1	150/218	55.8/72.9	**<0.0001**	**2.13(1.50**–**3.03)**	**2.99(2.03**–**4.43)**	
>60	46/53	39.7/41.7	70/74	60.3/58.3	0.840	0.92(0.55–1.53)	0.94(0.51–1.72)	
Sex								0.340
Male	143/106	43.5/30.0	186/247	56.5/70.0	**0.0003**	**1.79(1.31**–**2.45)**	**2.09(1.46**–**3.01)**	
Female	78/70	42.2/34.3	107/134	57.8/65.7	**0.006**	**1.39(0.92**–**2.10)**	**1.91(1.21**–**3.04)**	
Smoking status								0.060
Never	137/101	46.9/33.7	155/201	53.1/66.3	**0.0008**	**1.76(1.26**–**2.45)**	**1.90(1.35**–**2.68)**	
≤25 pack-year	31/47	48.4/27.5	33/124	51.6/72.5	**0.0024**	**2.48(1.37**–**4.49)**	**2.45(1.32**–**4.54)**	
>25 pack-year	18/21	48.6/33.9	19/41	51.4/66.1	0.145	1.85(0.81–4.25)	2.06(0.85–5.02)	
Missing	35/7	28.9/31.8	86/15	71.1/68.2	**–**	**–**	**–**	
Drinking status								0.360
Yes	29/38	50/28.4	29/96	50/71.6	**0.004**	**2.53(1.34**–**4.78)**	**2.66(1.36**–**5.19)**	
No	157/131	46.9/32.7	178/270	53.1/67.3	**<0.0001**	**1.82(1.35**–**2.45)**	**1.88(1.37**–**2.58)**	
Missing	35/7	28.9/31.8	86/15	71.1/68.2	–	–	–	
BMI(kg/m^2^)								0.240
<18.5	9/4	31.0/19.1	20/17	69.0/80.9	0.340	1.91(0.50–7.33)	1.62(0.37–7.05)	
18.5–24.0	117/93	46.2/34.1	136/180	53.8/65.9	**0.0044**	**1.67(1.17**–**2.37)**	**1.76(1.23**–**2.52)**	
>24.0	70/79	49.6/30.0	71/184	50.4/70.0	**<0.0001**	**2.30(1.51**–**3.50)**	**2.07(1.32**–**3.25)**	
Missing	25/0	27.5/0	66/0	72.5/0	–	–	–	
Ann Arbor Stage								**0.040**
I– II	137/176	42.5/31.6	185/381	57.5/68.4	**0.001**	**1.60(1.21**–**2.13)**	**1.89(1.38**–**2.59)**	
III–IV	84/176	43.7/31.6	108/381	56.3/68.4	**0.002**	**1.68(1.20**–**2.36)**	**2.28(1.51**–**3.46)**	
Subtype								0.490
B cell NHL	140/176	41.7/31.6	196/381	58.3/68.4	**0.002**	**1.55(1.17**–**2.05)**	**1.95(1.41**–**2.70)**	
DLBCL	100/176	42.9/31.6	133/381	57.1/68.4	**0.002**	**1.63(1.19**–**2.23)**	**2.01(1.41**–**2.85)**	
FL	29/176	55.8/31.6	23/381	44.2/68.4	**0.0004**	**2.73(1.53**–**4.85)**	**2.53(1.17**–**5.45)**	
T cell NHL	81/176	45.5/31.6	97/381	54.5/68.4	**0.0007**	**1.81(1.28**–**2.55)**	**2.22(1.49**–**3.30)**	
NK/T	11/176	21.6/31.6	40/381	78.4/68.4	0.137	0.59(0.30–1.19)	0.79(0.27–2.33)	

Statistically significant results (P<0.05) are highlighted in bold.

a P value of the comparison with a two-sided Chi-square test.

b ORs were obtained from logistic regression models with adjustment for age, sex, smoking status, alcohol use and BMI.

c P value of the heterogeneity assumption with a Chi-square-based Q-test.

## Discussion

Genetic polymorphisms in immune-related genes that regulate the immune and inflammation response may play an important role in the incidence of NHL [19], [20]. In this case-control study, we reported that *TNFAIP8* rs1045241C>T was significantly associated with an increased risk of NHL in a Chinese population. Stratified analyses revealed that subgroups of 40–60 years, non-smokers or light-smokers (≤25 pack-years), subjects with normal weight or overweight were more likely to have been diagnosed with NHL, especially the subtypes of DLBCL and FL. According to epidemiologic data about NHL in China, the main subtypes of lymphoma are DLBCL, FL and NK/T lymphoma, and other subtypes have a small sample size, and therefore we only analyzed the dominant subtypes of the cases. These results support the hypothesis that some polymorphism in inflammation and immune-related genes may be associated with risk of NHL and its common subtypes.

*TNFAIP8*, also known as *GG2-1*, *MDC-3.13*, *SCC-S2*, is located on chromosome 5 (5q23.1). It was first identified in a human head and neck squamous cell carcinoma (HNSCC) cell line [21]. Recently, it has been reported to be associated with oncogenesis, immunity, and inflammation in several studies [11], [12], [22]. *TNFAIP8* mRNA over-expression has been found in various malignant cell lines, such as breast cancer [23], non-small cell lung cancer [24], and esophageal squamous cell carcinoma [25]. Evidence showed that TNFAIP8 expression was upregulated by TNF-α induced NF-*κ*B pathway activation in cancer cell lines, which can inhibit caspase-8 and reduce apoptosis [26], [27]. Tumor necrosis factor-α induced protein 8-like 2 (*TIPE2*), a member of the TNFAIP8 family, was originally identified as a gene abnormally expressed in the inflamed spinal cord of mice with experimental autoimmune encephalomyelitis [12]. *TIPE2-*deficent mice were more likely to suffer from chronic inflammation diseases and multiple organ inflammation [12]. In humans, the abnormal expression of TIPE2 was associated with systemic autoimmunity [28], diabetic nephropathy [29], and hepatitis B [30]. These studies supported that TIPE2 plays an important role in maintaining immune homeostasis.

However, fewer studies have focused on the association between polymorphisms of *TNFAIP8* and NHL risk. The SNP *TNFAIP8* rs1045241C>T included in the present study is located on the 3′UTR of *TNFAIP8*, and the SNP function prediction shows that it may affect the microRNA-binding sites activity (http://snpinfo.niehs.nih.gov/snpfunc.htm). Some evidence has indicted that a genetic polymorphism in a microRNA target site influence transcriptional and post-transcriptional gene expression in cancers [31]. Though we found that rs1045241C>T was associated with the risk of NHL, the exact mechanisms of this relationship remained unknown. Further functional studies have been conducting to confirm our results.

In the present study, we did not find any significant association between the investigated polymorphisms of *TNF* rs1799964T>C, *LTA* rs1800683G>A, *IL-10* rs1800872T>G, *LEP* rs2167270G>A, *LEPR* rs1327118C>G and NHL risk in a Chinese population. However, many pieces of evidence have shown that other SNPs in these genes like *TNF*–308 G>A (rs1800629), *LTA* 252A>G (rs909253), *IL10*–3575T>A (rs1800890), *LEPR* 233Q>R (rs1137101) increased the risk of NHL, particularly DLBCL in non-Hispanic white populations [9], [14], [15], [32], [33]. Our studies did not cover these SNPs because their MAF was <5% in CHB population according to Hapmap database (http://hapmap.ncbi.nlm.nih.gov/). But Xiao et al. reported that no association between *TNF*–308, *LTA* 252 polymorphisms and histological subtypes, disease stages in Chinese NHL patients [34]. We believe that this inconsistency may be due to ethnic and demographic differences between Chinese populations and white populations as well as the smaller sample size that may also have led to insufficient statistical power. So our results need to be replicated in additional studies in different populations with large sample sizes.

Fewer studies reported an association between *LEP* 19 A>G, *LEPR* 233Q>R and risk of NHL in Chinese populations, though some previous published studies revealed that obesity was a risk factor of NHL in developed countries [35]. Obesity may play the role of inducing lymphomagenesis by the co-effects of *LEP* 19 A>G, *LEPR* 233Q>R polymorphisms and immune dysfunction [14], [15], [36]. Inconsistently, we found the NHL cases were more likely to have lower BMI compared with the cancer-free controls (*P*<0.0001). One possible reason may due to ethnic difference in BMI and its contribution to lymphoma. It is likely that populations of developing and developed countries have different body size, and Asians have lower BMI than Westerners. Thus the Chinese standard of obesity was applied to the subjects in this study, but obesity did not appear closely related to lymphomagenesis. However, another possibility was that obesity may not a direct risk factor for NHL in Chinese populations. There may be some intricate interaction among obesity, diet, hormone, genetics, and susceptibility to NHL but the present study did not have enough power to detect such interactions. Further larger, transcontinental studies are needed to fully explore and elaborate the extent of such interactions.

In the stratified analyses, the risk effect of the *TNFAIP8* rs1045241 CT/TT genotype was more evident in subgroups of 40–60 year-old, non-smoker or light-smoker, and normal BMI or overweight. Stratifying by subtypes and stage, the risk effect of rs1045241 CT/TT genotype was evident for both B and T cell lymphoma, especially for DLBCL and FL as well as later Ann Arbor Stage of ?–?. But we noticed that after verification of homogeneity assumption, only age and stage showed marginally statistical significance. So, there was no strong evidence of an effect between the selected covariates and NHL risk. The exact association between selected covariates and NHL risk needs to be further investigated by large studies. Up to now, there is still inconsistence about the effect of smoking, alcohol use, and BMI on NHL risk, and the mechanisms are multiple. Wang et al. once reported that autoimmune conditions, obesity, and later birth order could contribute to lymphomagensis through an alteration of the proinflammatory pathway, specifically involving common genetic variants in *TNF* and *IL-10* [8]. Given the results of our study, NHL development may be of complex process that includes a great number of events. A single factor would have a limited effect on the susceptibility. Once the exposure of environmental factors was accumulated to a certain level, alteration took place in immune and proinflammatory pathways, and genetic variants or other unknown events may also participate in the process. On the other hand, our study was not large enough, and possible selection bias may exist due to a higher-than expected non-response rate in case group. Well-designed large studies are needed to test the hypothesis that *TNFAIP8* polymorphisms may influence NHL susceptibility, particularly by the possible interactions with environmental risk factors.

Several limitations exist in the present study. First, the sample size was not large enough, so selection bias may be inevitable, and the study did not provide enough statistical power to detect gene-gene and gene-environment interaction. Second, we selected only one functional SNP for each candidate gene, which restricted further analysis to identify other potentially important associations. Moreover, different histological subtypes of NHL, such as DLBCL, FL, and NK/T cell lymphoma may have different risk factors and etiologies. We did not have enough information about environmental exposure from NHL patients, such as use of hair dye, family history of NHL, occupation and EB-virus infection to be included in stratified analyses.

In summary, we identified *TNFAIP8* rs1045241C>T to be associated with risk of NHL in a Chinese population, particularly DLBCL and FL. To the best of our knowledge, this is the first study to report a *TNFAIP8* polymorphism associated with NHL susceptibility in a Chinese population, which provides additional evidence that inflammation and immune-related genetic polymorphisms play an important role in lymphomagenesis. Further large-scale well-designed population-based studies are needed to validate our findings, define the high-risk population and deepen our understanding of NHL pathogenesis.
